# Genetic Variants Are Not Rare in ICD Candidates with Dilated Cardiomyopathy: Time for Next-Generation Sequencing?

**DOI:** 10.1155/2019/2743650

**Published:** 2019-04-24

**Authors:** Alexandra Sousa, Paulo Canedo, Manuel Campelo, Brenda Moura, Sérgio Leite, Márcia Baixia, Adriana Belo, Francisco Rocha-Gonçalves, José Carlos Machado, José Silva-Cardoso, Elisabete Martins, FATIMA Investigators

**Affiliations:** ^1^Department of Medicine, Faculty of Medicine, Alameda Prof. Hernâni Monteiro, 4200-319 Porto, Portugal; ^2^Cintesis-Center for Research in Health Technologies and Services, Rua Dr. Plácido da Costa, 4200-450 Porto, Portugal; ^3^Department of Cardiology, Santa Maria Maior Hospital, Campo da República, Apartado 181, 4754-909 Barcelos, Portugal; ^4^i3S‐Institute for Research and Innovation in Health, Rua Alfredo Allen, 4200-135 Porto, Portugal; ^5^Department of Cardiology, Centro Hospitalar Universitário de São João, E.P.E., Alameda Prof. Hernâni Monteiro, 4200-319 Porto, Portugal; ^6^Department of Cardiology, Hospital das Forças Armadas - Pólo Porto, Av. Da Boavista, 4050-113 Porto, Portugal; ^7^Department of Cardiology, Alto Ave Hospital Center–Guimarães Unity, Rua dos Cutileiros 114, Creixomil, 4835-044 Guimarães, Portugal; ^8^National Center for Data Collection in Cardiology, Rua de Olivença n° 11, 7° Piso, Sala 701, 3000-306 Coimbra, Portugal

## Abstract

**Background:**

Sudden cardiac death (SCD) risk stratification in dilated cardiomyopathy (DCM) has been based on left ventricular ejection fraction (LVEF), even though SCD may occur with LVEF > 35%. Family history of unexplained SCD, especially in the young, raises concern about potential inheritable risk factors. It remains largely unknown how genetic tests can be integrated into clinical practice, particularly in the selection of implantable cardioverter defibrillator (ICD) candidates. We aimed to assess the diagnostic yield of genetic testing in DCM patients with a class I recommendation for ICD implantation, based on current guidelines.

**Methods:**

We included ambulatory stable adult patients with idiopathic or familial DCM with previously implanted ICD. Molecular analysis included 15 genes (*LMNA*, *MYH7*, *MYBPC3*, *TNNT2*, *ACTC1*, *TPM1*, *CSRP3*, *TCAP*, *SGCD*, *PLN*, *MYL2*, *MYL3*, *TNNI3*, *TAZ*, and *LDB3*) using next-generation sequencing.

**Results:**

We evaluated 21 patients, 12 (57%) males and 9 (43%) with familial DCM, including 3 (14%) with a family history of premature unexplained SCD. Mean age at DCM diagnosis was 40 ± 2 years, and mean age at ICD implantation was 50 ± 12 years. LVEF was 27 ± 9%, and LV end-diastolic diameter was 65 ± 7 mm. Genetic variants were found in six (29%) patients, occurring in 5 genes: *TPM1*, *TNNT2*, *MYH7*, *PLN*, and *MYBPC3*. The majority were classified as variants of uncertain significance. Family history of SCD was present in both patients with *PLN* variants.

**Conclusion:**

In patients with DCM and ICD, genetic variants could be identified in a significant proportion of patients in several genes, highlighting the potential role of genetics in DCM SCD risk stratification.

## 1. Introduction

Sudden cardiac death (SCD) is responsible for a significant number (30%) of deaths in patients with DCM [1, 2], and implantable cardioverter defibrillator (ICD) devices can reduce this risk [3].

Currently, the preferred clinical tool to estimate SCD risk and decide about ICD implantation for primary prevention is the presence of LV ejection fraction (LVEF) value ≤ 35%, after heart failure medical therapy uptitration [1]. However, risk prediction of SCD in DCM patients is far from being ideal. Most of the episodes of SCD continue to occur despite LVEF values higher than 35% and, most recently, the DANISH trial showed no long-term mortality benefit for prophylactic ICD implantation, in patients with symptomatic nonischemic heart failure with reduced ejection fraction [4].

The diversity of etiologies and heterogeneity of arrhythmogenic mechanisms can explain the difficulty in predicting SCD risk in DCM patients.

A frequently unrecognized subgroup of patients is that with genetic DCM. Genetic factors have been associated with higher arrhythmic risk [5], but in most patients, genetic tests are not performed before ICD implantation. According to Heart Rhythm Society (HRS) and the European Heart Rhythm Association (EHRA), genetic screening is recommended when there is evidence of DCM and significant cardiac conduction disease and/or a family history of unexplained premature sudden cardiac death. These characteristics are considered as “red flags” for *LMNA* and *SCN5A* associated disease [6].

Nowadays, molecular diagnosis is performed using next-generation sequencing (NGS) that allows several genes to be sequenced simultaneously, with decreased costs and turnaround times. This technical advantage is particularly useful in DCM, a disease with a high genetic and allelic heterogeneity, with 60 to 90% of causal variants found in only single families [7]. The main difficulty related to the higher yield of genetic variants obtained with NGS is the ascription of pathogenicity for most variants [8]. Crossing data from different genetic databases, containing clinical and laboratory information of DCM patients, is of utmost importance for this purpose, and also to highlight the usefulness of genetic tests in arrhythmic risk prediction algorithms.

In our study, we aimed to assess the diagnostic yield of genetic analysis using NGS in DCM patients with *a priori* class I recommendation for ICD implantation, based on the European Society of Cardiology guidelines [1].

## 2. Materials and Methods

### 2.1. Population

A multicentric study of adult unrelated ambulatory patients (stable in NYHA I or II for at least 6-month) with idiopathic DCM (age ≤ 50 years) or familial DCM (irrespective of age), with a class I indication for ICD implantation [9], was included in the study. DCM was diagnosed according to the Working Group on Myocardial and Pericardial Disease of the ESC criteria [10]. DCM was considered idiopathic in the absence of evidence for a secondary or acquired cause of the disease. Familial disease was established when idiopathic DCM was present in more than one family member or when unexplained SCD occurred in any first-degree relative under the age of 35 years [11, 12]. Nonfamilial disease was considered in the remaining cases, after a complete and detailed questionnaire on the patient family history and clinical, electrocardiographic, and echocardiographic evaluation of first-degree relatives.

This study is a subanalysis of FATIMA (Portuguese study of familial dilated cardiomyopathies) and general clinical assessment, and cardiological investigations as well as exclusion criteria (any possible secondary or acquired cause of the disease) have been previously described [11]. We also excluded patients considered for or on the waiting list for heart transplantation. Cardiovascular events, such as cardiovascular death and ventricular arrhythmic episodes, were collected in each clinical visit by anamnesis and consult of clinical records.

### 2.2. Next-Generation Sequencing

A peripheral blood sample was obtained for molecular analysis from each patient. Screening of variants in a panel of 15 genes—lamin A/C (*LMNA*), beta-myosin heavy chain (*MYH7*), myosin-binding protein C (*MYBPC3*), troponin-T (*TNNT2*), alpha-actin (*ACTA1*), alpha-tropomyosin (*TPM1*), cysteine-rich protein 3 (*CSRP3*), titin-cap (*TCAP*), sarcoglycan delta (*SGCD*) and phospholamban (*PLN*), regulatory and essential light chains (*MYL2* and *MYL3*), troponin-I (*TNNI3*), tafazzin (*TAZ*), and ZASP/Cypher (*LDB3*)—was performed in all patients, using NGS with a minimum of 30-fold coverage and guaranteed 100% horizontal coverage for the coding sequencing and flanking exon/intron regions. Sanger sequencing was used to validate the identified variants and to provide additional coverage for regions of the panel with less than 30-fold coverage. Genes were selected, and the targeted gene panel was designed in 2010, based on previously identified variants in DCM patients at the time.

For the screening of variants in the selected genes, primers were designed for all coding exons, covering exon/intron boundaries using freely available software Primer3. No known variants were present in the primer sequences (dbSNP built 130). A multiplex PCR-based strategy was used to reduce the number of amplification reactions (primer sequences are provided upon request). Multiplex PCR reactions were performed following the QIAGEN Multiplex PCR Kit protocol (Qiagen, Hilden, Germany).

#### 2.2.1. Library and Template Preparation

**Figure 1:**
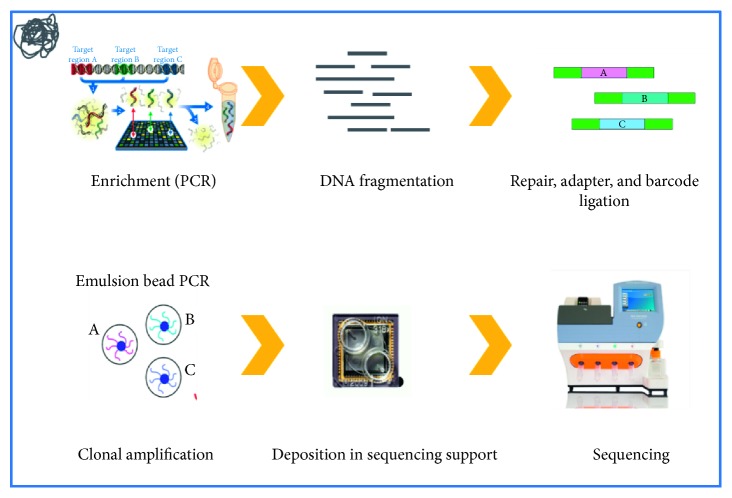
Molecular analysis: library and template preparation.

Sample quality of patient genomic DNA was evaluated by gel electrophoresis and quantified using Qubit dsDNA HS Assay Kit (Life Technologies). A total of 50 ng of genomic DNA was used in each multiplex PCR reaction. Library preparation was performed using the protocol Ion Xpress Plus gDNA and amplicon library preparation (PN4471989 Rev. C). Library quantification was performed using the Qubit dsDNA HS assay. All libraries were diluted to the same concentration and pooled to ensure an equal representation of the different samples. The diluted and combined libraries were subjected to amplification by emulsion PCR using Ion Template OT 2 200 Kit (Life Technologies) on an Ion OneTouch 2 instrument (Life Technologies) according to the manufacturer's protocol. Enrichment of template ion sphere particles was performed using the Ion OneTouch 2 enrichment system (Life Technologies) (Figure 1).

#### 2.2.2. Semiconductor Sequencing and Data Analysis

**Figure 2:**
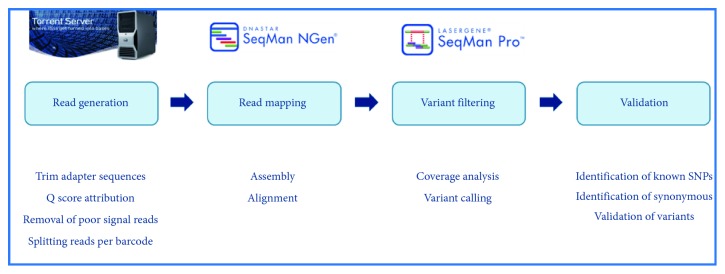
Molecular analysis: bioinformatic pipeline.

Sequencing was carried out on an Ion PGM system based on semiconductor technology. The Ion Sequencing Kit v2.0 (Life Technologies) was used to perform sequencing runs, following the manufacturer's recommended protocols. Data from the PGM runs were processed using the Ion Torrent platform-specific pipeline software Torrent Suite v4.2 (Life Technologies) to generate sequence reads, trim adapter sequences, filter and remove poor signal reads, and split the reads according to the barcode. Reads assembly was performed with SeqMan NGen v4.1 (DNAStar, Madison, Wisconsin) using the FastQ files containing sequence reads and the template references adjusted for the covered amplicons. SeqMan Pro v10 (DNAStar) was used as a postassembly analysis tool for the analysis of overall amplicon coverage, individual base depth of coverage (only considered if coverage has a value of, at least, 30), and variant identification. A filter for the coding sequencing variants (CDS) was applied to the SNP report, for each case. All rare nonsynonymous variants identified (MAF < 0.01) were independently confirmed by Sanger sequencing (Figure 2).

From there, each variant was interpreted and pathogenicity was assessed in accordance with the American College of Medical Genetics and Genomics [8], by comparisons with populational and disease databases, where genetic variants have been previously described, and predictive bioinformatics models, familial segregation analysis, and functional studies when available. They were classified in “pathogenic,” “likely pathogenic,” “of uncertain significance,” “likely benign,” and “benign.”

### 2.3. Ethical Approval

The study protocol conforms to the ethical guidelines of the Declaration of Helsinki, 1975, as reflected in a priori approval by the local ethics committees for human research. Informed consent was obtained from each patient.

## 3. Results

We evaluated 21 patients, 12 (57%) male, 9 (43%) with familial DCM, including 3 (14%) with a family history of premature unexplained SCD. Mean age at DCM diagnosis was 40 ± 12 years, and the mean age at ICD implantation was 50 ± 12 years. Echocardiographic LVEF was 27 ± 9%, LV end-diastolic diameter 65 ± 7 mm, and 7 (33%) patients also presented right ventricular dysfunction. Cardiac magnetic resonance (CMR) was only performed in seven (33%) patients, since the previous implantation of ICD precluded its execution. In these patients, LVEF was 28 ± 10%, LV end-diastolic volume was 144 ± 26 mL/m^2^, and two patients (29%) presented late gadolinium enhancement. Left bundle branch block was present in ten (48%) patients, and six patients (35%) had documentation of nonsustained ventricular tachycardia on the 24-holter recording. No patient had evidence of second and/or third atrioventricular block. Two patients had elevated plasma creatinine kinase. Further patients' characterization is displayed in Table 1.

**Figure 3:**
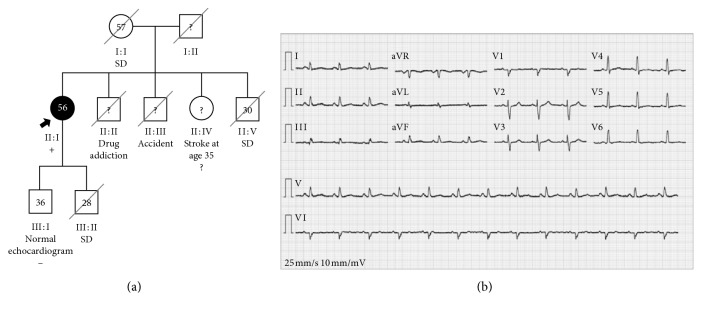
Pedigree (a) and electrocardiogram (b) of the patient with the p.Pro21Thr phospholamban variant. The electrocardiogram reveals sinus rhythm and a QRS width of 101 ms. Squares: male; circles: female; dark symbol: dilated cardiomyopathy; ±symbols: presence/absence of *PLN* variant; numbers inside the symbols: current age; SD: sudden death; “?”: inexistent information or availability for clinical/genetic assessment.

Rare genetic variants were found in six (28%) of these patients, occurring in 5 different genes: *TPM1*, *TNNT2*, *MYH7*, *PLN*, and *MYBPC3*. Tables 2 and 3 show the clinical characteristics of those patients and genetic variant classification, respectively. The pedigree and the ECG of a *PLN* carrier are illustrated in Figure 3.

The *TPM1* variant, c.758T > C p.(Ile253Thr), was absent from controls (ExAC; 1000 genomes) and from disease-specific databases. *In silico* analysis predicts it to be disease-causing and probably damaging to the protein structure/function. To our knowledge, this residue is conserved throughout evolution. No other variants have been reported in association with disease in nearby codons.

The *TNNT2* variant, c.517C > T p.(Arg173Trp), was absent from controls (ExAC; 1000 genomes), and it has four entries in ClinVar as pathogenic/likely pathogenic. This variant is a nonconservative amino acid substitution, which is likely to impact secondary protein structure as these residues differ in polarity, charge, size, and/or other properties, which is supported by *in silico* analysis. This substitution occurs at a position in which amino acids with similar properties to arginine are tolerated across species. Another variant in the same residue (Arg173Gln) as well as variants in nearby residues (Glu163Lys, Ala172Ser, and Ser179Phe) have been reported at HGMD in association with cardiomyopathy. Cardiomyocytes generated from induced pluripotent stem cells from patients of a family harboring Arg173Trp exhibited altered Ca^2+^ handling and impaired myofilament regulation [22]. In [23], this variant segregated with all affected family members in two families. Also, a patient with a borderline phenotype did not harbor the variant and did not progress in over more than 20 years of follow-up [23].

The *MYH7* variant, c.5623G > T p.(Val1875Phe), was absent from controls (ExAC; 1000 genomes) and from disease-specific databases. *In silico* analysis predicts it to be disease-causing and probably damaging due to the protein structure/function. To our knowledge, this is a conservative amino acid change, and this residue is conserved throughout evolution. No other variants have been reported in association with disease in nearby codons. A variant in the same residue (Val1875Ala) has an entry in ClinVar, as uncertain significance.

As for the *PLN* variants, only one missense variant is reported as pathogenic in ClinVar (Arg9Cys). The variant c.61C > A p.(Pro21Thr) is present in very low frequency in controls (ExAC 0.00006 (A)) and, despite being a nonconservative change, the *in silico* analysis does not have concordant results. The variant c.23C > T p.(Thr8Ile) was absent from controls (ExAC; 1000 genomes) and from disease-specific databases. *In silico* analysis predicts it to be disease-causing and probably damaging to the protein structure/function. A variant in the same residue (Thr8Pro) has an entry in ClinVar, as uncertain significance. Also, despite being a nonconservative change, the *in silico* analysis does not have concordant results.

The *MYBPC3* variant c.1226 + 6T > C is present in very low frequency in controls (ExAC 0.00006 (G)), and it has one entry in ClinVar as uncertain significance. This variant is not located in a canonical splice site and therefore is a rare intronic change with uncertain impact on splicing.

When comparing patients with and without a rare variant, there were no significant differences in main demographic, clinical, and electrocardiographic characteristics, although those with a positive molecular study presented higher LVEF (34 ± 7% vs. 24 ± 8%, *p*=0.024) (Table 4).

After a mean follow-up of 21 ± 8 months (range 5–30), 3 (14%) patients experienced adverse events. Two patients were hospitalized because of heart failure, and one of them ultimately died from pump failure; the other presented ventricular arrhythmia, namely, a nonsyncopal monomorphic ventricular tachycardia (eight months later underwent heart transplantation). The patient who died from heart failure had the *MYBPC3* variant c.1226 + 6T > C, and the remaining had a negative molecular study.

## 4. Discussion

In our DCM patients with a class I indication for ICD implantation, using NGS methodology for genetic analysis, we found a non-negligible number of variants in different genes. Most of the identified variants, although predicted to be functionally significant, were classified as of uncertain significance mainly because the evaluation of patients' families did not allow conclusions about segregation and because half of them were novel variants, not previously described (*MYH7* p.(Val1875Phe), *TPM1* p.(Ile253Thr) and *PLN* p. (Thr8Ile)). Only the *TNNT2* variant p.(Arg173Trp) was previously described in DCM families [20, 23]. The *MYBPC3* variant c.1226 + 6T > C was previously described in association with hypertrophic cardiomyopathy [20].

In general, there were no major clinical differences between patients with or without genetic variants in the analyzed genes. Interestingly, patients with positive molecular study presented a higher LVEF. This could be related to a higher proportion of patients with ICD implanted in a context of secondary prevention of sudden cardiac death or we could hypothesize that the involved genes might be somehow related to left ventricular reverse remodelling as has been shown recently for titin [24] and other genes [25]. Also, the presence of a genetic variant did not influence the occurrence of an adverse event, although the small number of participants and events limits our conclusions. Even so, these results are interesting, as they put in perspective the potential role of genetics in SCD risk stratification.

DCM genetics has been previously assessed in patients with life-threatening arrhythmias and analyzed in few larger genotype-phenotype correlation studies, including the INTERHEART study, where 27% received ICD implantation [26]. SCD risk has been particularly associated with *LMNA* variants, but also in other genes, such as *PLN*, *SCN5A*, and *FLNC* and some desmosomal genes [5, 27, 28].

In our patients, we did not find any variant in the *LMNA* gene, but this could be partially related to the fact that no patient presented evidence of atrioventricular conduction block on ECG and no patient considered for or on the waiting list for heart transplantation has been included (even though almost half of our patients presented left bundle branch block and 35% presented nonsustained ventricular tachycardia, features also common in *LMNA* pathogenic variants carriers with DCM) [5]. On the other hand, two of our three patients with a family history of SCD presented *PLN* gene variants, one of which is a novel variant (Thr8Ile).

Phospholamban is a key protein involved in calcium metabolism, regulating SERCA Ca^2+^ affinity at the sarcoplasmic reticulum. Mutations in *PLN* can be identified in 2% of DCM patients [27], and variants in this gene have been particularly associated with arrhythmogenic cardiomyopathies, including right ventricular dysplasia with predominant LV involvement [29, 30]. The arrhythmogenic mechanisms of *PLN* variants are largely unknown, but probably they are variable and depend on the variant itself [31]. Mechanisms involving the irreversible superinhibition of sarcoplasmic reticulum ATPase due to the deletion of arginine 14 in *PLN* gene (Arg14del) have been described in familial DCM with premature sudden cardiac death [32, 33]. Recently, a new hypothesis involving interference with intercalated disc remodelling has been proposed [34]. In both of our patients with *PLN* variants, arrhythmogenicity could essentially be inferred from family history, particularly in the case 4, presented in Figure 3. This proband is a woman with the diagnosis of DCM at the age of 47, with mild symptoms of heart failure and documented runs of nonsustained ventricular tachycardia, with history sudden death in three first-degree relatives. Of note, her ECG did not present any distinctive characteristic (only a slight prolongation of the duration of the QRS), unlike the ECG of *PLN* p.(Arg14del) carriers, which presented strikingly attenuated R amplitudes, independent of the presence of echocardiographic abnormalities [32, 33]. This particular eletrocardiographic characteristic is apparently related with ventricular fibrosis [32, 33]. Unfortunately, CMR was not performed in any of our patients before device implantation, an exam that could have added some other important details in phenotypic characterization [35].

In order to improve the knowledge about arrhythmogenesis in DCM and the clinical usefulness of genetics in SCD risk stratification, it would be interesting to consider molecular diagnosis as an integrant part of all DCM patients' evaluation, as recommended in recent guidelines on genetic evaluation on cardiomyopathies [36]. This would allow the creation of clinical databases of different sequence variations with information about the clinical profile (including age of onset, disease course, and any unique features and outcomes) along with the variant identified, increasing insights into genotype-phenotype correlations, in a larger group of patients, and permitting continued and extended follow-up of genetically characterized DCM patients.

## 5. Limitations

The major limitation of our work is the small number of patients and the restricting panel of analyzed genes, particularly the noninclusion of titin that accounts for up to 20–25% of all DCM cases [27], as well as other “arrhythmogenic” genes (when the study was designed, the panel of genes was chosen, given the published overall percentage of dilated cardiomyopathy cases caused by pathogenic variants in each gene, being one of the broader panels available at the time for cardiomyopathies). Even though this study refers to a rather small group of patients and the number of genes analyzed is limited, our results may be a contribution to the better understanding of electric burden in patients with dilated cardiomyopathy who are candidates to ICD implantation.

Another important limitation refers to our inability to differentiate the setting of ICD implantation (primary vs. secondary prevention). Additionally, we did not include patients with ICD considered for or on the waiting list for heart transplantation, because we considered that they represent another particular subgroup of patients, with end-stage disease, dominated by the progressive LV dysfunction.

These limit the power of our conclusion that might not be extrapolated to other scenarios and should, therefore, be considered hypotheses generating.

## 6. Conclusions

Arrhythmogenesis in DCM is complex and derives from different mechanisms. The role of all possible contributing mechanisms, including fibrosis, inflammation, or genetics, is important, considering the actual difficulty in stratifying SCD risk. Our findings contribute to the better understanding of electric burden in DCM patients and reinforce, in part, the potential usefulness of genetic testing to improve the selection of candidates for ICD implantation.

Until the role of molecular diagnosis in the decision of ICD implantation is well established, the criteria to proceed to genetic testing maybe should not be too restrictive, confined to the presence of familial events or a particular phenotypic characteristic.

The use of broader gene panels in larger cohorts and collection of extended follow-up data are essential to achieve more definitive conclusions about the clinical usefulness of genetic tests in SCD risk stratification in DCM patients.

## Figures and Tables

**Table 1:** Patients' characteristics.

Characteristics	DCM patients (*n*=21)
Gender, *n* (%)	
Male	12 (57)
Female	9 (43)

Age at diagnosis, years (mean ± SD)	40 ± 12

Age at ICD implantation, years (mean ± SD)	50 ± 12

Familial DCM, *n* (%)	9 (43)
Idiopathic DCM, *n* (%)	12 (57)

Family history, *n* (%)	
Heart failure-related death	7 (33)
Sudden death	3 (14)

Clinical presentation, *n* (%)	
Heart failure symptoms	7 (33)
Syncope or arrhythmia	3 (14)
Chest pain	5 (24)
Others^*∗*^	1 (5)

NYHA functional class, *n* (%)	
I	6 (28)
II	13 (62)
III	1 (5)
IV	1 (5)

Previous hospitalizations, *n* (%)	13 (62)
Heart failure-related	10 (48)
Arrhythmic causes	7 (33)
Others^†^	1 (5)

ECG data, *n* (%)	
AF/atrial flutter	4 (19)
LBBB	10 (48)
PVC	5 (24)
NSVT	6 (35)

Echocardiographic data	
LVEDD (mm) (mean ± SD)	65 ± 7
LVEF (%) (mean ± SD)	27 ± 9
RV impairment, *n* (%)	7 (33)

CMR data, *n* (%)	7 (33)
LVEDV (mL/m^2^) (mean ± SD)	144 ± 26
LVEF (%) (mean ± SD)	28 ± 10
LGE, *n* (%)	2 (28)

Medical therapy, *n* (%)	
ACEI/ARB	19 (90)
Beta-blockers	18 (86)
MRA	14 (67)
Diuretic	12 (57)
Digoxin	6 (28)
Ivabradine	2 (10)

^*∗*^Asymptomatic left ventricular dysfunction diagnosed on medical sportive examination. ^†^Elective hospitalization for atrial flutter ablation. ACEI: angiotensin converting enzyme inhibitor; AF: atrial fibrillation; ARB: angiotensin II receptor blocker; CMR: cardiac magnetic resonance; DCM: dilated cardiomyopathy; ECG: electrocardiogram; ICD: implantable cardioverter defibrillator; LBBB: left bundle branch block; LGE: late gadolinium enhancement; LVEDD: left ventricular end-diastolic diameter; LVEDV: left ventricular end-diastolic volume; LVEF: left ventricular ejection fraction; MRA: mineralocorticoid receptor antagonist; NSVT: nonsustained ventricular tachycardia; NYHA: New York Heart Association; PVC: premature ventricular contraction; RV: right ventricle; SD: standard deviation.

**Table 2:** Clinical characteristics of patients with genetic variants.

Case	Gender	Age	Age at diagnosis	NYHA class	Etiology	ECG-rhythm disturbances	ECG-conduction disturbances	LVEF (%)	LVED (mm)	Affected gene
1	Female	40	17	II	IDCM	SR	—	38	61	*TPM1*
2	Male	53	38	II	IDCM	SR	LBBB	38	63	*TNNT2*
3	Male	55	53	II	IDCM^*∗*^	SR	LBBB	39	56^†^	*MYH7*
4	Female	56	47	I	FDCM^‡^	SR	—	37	68	*PLN*
5	Male	53	41	II	FDCM^‡^	SR	—	33	62	*PLN*
6	Female	72	58	II	FDCM	AF	LBBB	20	71	*MYBPC3*

FDCM: familial dilated cardiomyopathy; IDCM: idiopathic dilated cardiomyopathy; LBBB: left bundle branch block; LVED: left ventricular end-diastolic diameter; LVEF: left ventricular ejection fraction; SR: sinus rhythm; ^*∗*^serum creatine kinase elevation; ^†^left ventricular hypertrabeculation/noncompaction; ^‡^sudden death in relatives.

**Table 3:** Classification of genetic variants (hg 19).

Gene	Transcript	NM (NCBI)	Genomic location	Nucleotide change	Amino acid change	MAF ExAC	dbSBP	SIFT [13]	Mutation Taster [14]	Polyphen-2 [15]	ACMG/AMP final classification and criteria^*∗*^
*TPM1*	ENST00000288398	NM_000366.5	Chr15:63354830	c.758T > C^#^	p.Ile253Thr	—	—	Deleterious	Disease causing	Possibly damaging	VUS (PM2, PP3)
*TNNT2*	ENST00000367318	NM_001001430.2	Chr1:201332477	c.517C > T	p.Arg173Trp	—	rs727503512	Deleterious	Disease causing	Probably damaging	Likely pathogenic (PS4, PP3, PP1, PP5, PP4)
*MYH7*	ENST00000355349	NM_000257.3	Chr14:23414039	c.5623G > T^#^	p.Val1875Phe	—	—	Deleterious	Disease causing	Probably damaging	VUS (PM2, PP3)
*PLN*	ENST00000357525	NM_002667.3									
Case 4			Chr6:118880145	c.61C > A	p.Pro21Thr	0.00005779	rs397516786	Tolerated	Disease causing	Probably damaging	VUS (PP3)
Case 5			Chr6:118558944	c.23C > T^#^	p.Thr8Ile	—	—	Deleterious	Disease causing	Probably damaging	VUS (PM2)
*MYBPC3*	ENST00000545968	NM_000256.3	Chr11:47364805	c.1226 + 6T > C^†^	—	0.00006195	rs397515892	—	—	—	VUS

ACMG/AMP: American College of Medical Genetics and Genomics/Association for Molecular Pathology [8]. MAF: minor allele frequency. ^*∗*^Evidence for variant classification: PS, pathogenic strong; PM, pathogenic moderate; PP, pathogenic supporting; the numbering within each category does not convey any differences of weight and refer the different criteria: PS4, the prevalence of the variant in affected individuals is significantly increased compared with the prevalence in controls; PP1, cosegregation with disease in multiple affected family members in a gene is definitively known to cause the disease; PP3, multiple lines of computational evidence support a deleterious effect on the gene or gene product; PP4, patient's phenotype or family history is highly specific for a disease with a single genetic etiology; PP5, reputable source recently reports variant as pathogenic, but the evidence is not available to the laboratory to perform an independent evaluation; PM2, the variant is absent from (or below the expected carrier frequency if recessive) a large general population or a control cohort (>1000 individuals), and the population is race-matched to the patient harboring the identified variant. ^#^Novel variant (not present in Exome Aggregation Consortium (ExAC) [16], Exome Sequencing Project (ESP) (http://evs.gs.washington.edu/EVS), 1000 Genomes (1 KG) [17], Single Nucleotide Polymorphism (dbSNP) [18], The Human Gene Mutation Database (HGMD) [19], ClinVar [20], and Leiden Open Variation Database (LOVD)) [21]. ^†^This variant has probably no impact on splicing. VUS: variant of uncertain significance.

**Table 4:** Patients' characteristics according to molecular study results.

Patient characteristics	No variant (*n*=15)	Variant-positive (*n*=6)	*p* value
Age at *dx*, years (mean ± SD)	40 ± 11	42 ± 14	0.633
Age at ICD implantation, years (mean ± SD)	50 ± 12	51 ± 13	0.872
Male, *n* (%)	9 (60)	3 (50)	1.000
Familial cases, *n*(%)	6 (40)	3 (50)	1.000
Family history, *n* (%)			
Sudden cardiac death	1 (7)	2 (33)	0.184
Previous hospitalizations, *n* (%)	9 (64)	4 (67)	1.000
Heart failure-related	8 (57)	2 (33)	0.628
Arrhythmic causes	4 (29)	3 (50)	0.613
NYHA, *n* (%)			
>I	10 (67)	5 (83)	0.802
ECG data, *n* (%)			
LBBB	7 (47)	3 (50)	1.000
Atrial fibrillation	3 (20)	1 (17)	1.000
NSVT	4 (36)	2 (33)	1.000
PVC	5 (33)	0 (0)	0.262
Echocardiogram data			
LVEDD (mm) (mean ± SD)	65 ± 7	63 ± 6	0.503
LVEF (%) (mean ± SD)	24 ± 8	34 ± 7	0.024
RV impairment, *n* (%)	4 (31)	3 (50)	0.617

ICD: implantable cardioverter defibrillator; LBBB: left bundle branch block; LVEDD: left ventricular end-diastolic diameter; LVEF: left ventricular ejection fraction; NSVT: nonsustained ventricular tachycardia; RV: right ventricle; SD: standard deviation.

## Data Availability

The data used to support the findings of this study are available from the corresponding author upon request.
